# Severe ocular sequelae of congenital toxoplasmosis: huge macular scar

**DOI:** 10.11604/pamj.2015.20.233.5097

**Published:** 2015-03-12

**Authors:** Fadoua Zahir, Meriem Abdellaoui, Samar Younes, Idriss A Benatiya, Hicham Tahri

**Affiliations:** 1 Ophthalmology Service, CHU Hassan II, Fes, Morocco

**Keywords:** Congenital toxoplasmosis, retinochoroiditis, sequelae

## Abstract

Retinochoroiditis is the most common ocular manifestation of congenital toxoplasmosis, but other associated ophthalmological pathologies can also occur. Ophthalmologists are rarely able to distinguish between toxoplasmic retinochoroiditis due to infection acquired before or after birth, unless other clinical or serological indications are present. This article reports a case of a 3-year-old boy with abnormalities suggestive of congenital toxoplasmosis. The clinical and complementary examinations are discussed. The education of pregnant women is crucial for the prevention of congenital toxoplasmosis. Awareness of antenatal and postnatal presenting signs and symptoms is important for clinicians, because early diagnosis and treatment may minimize sequelae. Untreated, the majority of affected infants will develop chorioretinitis, deafness and/or neurological symptoms.

## Introduction

Retinochoroiditis is the most common ocular manifestation of congenital toxoplasmosis, but other associated ophthalmological pathologies can also occur [1]. Ophthalmologists are rarely able to distinguish between toxoplasmic retinochoroiditis due to infection acquired before or after birth, unless other clinical or serological indications are present [2]. Awareness of antenatal and postnatal presenting signs and symptoms is important for clinicians, because early diagnosis and treatment may minimize sequelae [3].

## Patient and observation

**Figure 1:**
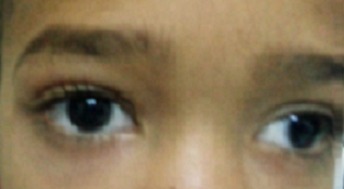
Exotropia for the left eye

A 3 year-old boy of a not followed pregnancy presented with an 8 months history of exotropia of the left eye (Figure 1). Cycloplegic refraction was -0.25 and 0.00 in the right and left eyes respectively.

**Figure 2:**
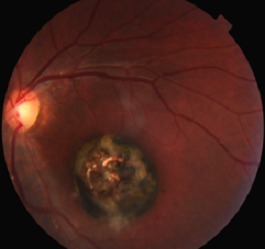
Macular scar of about 3 papillary diameters

An initial objective assessment of the visual function showed a best corrected visual acuity of 12/10 in the right eye and Counting fingers at 1m in the left eye. Examination of the anterior segment was unremarkable in both eyes. Fundoscopy revealed macular scar of about 3 papillary diameters (Figure 2).

**Figure 3:**
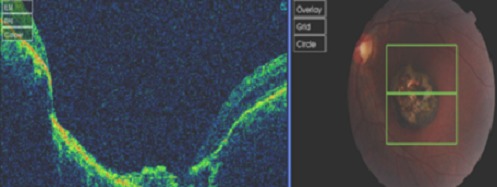
Optical coherence tomography (OCT) of the macula reveals a disorganization of retinal architecture related to a sequelae of retinochoroiditis

The optical coherence tomography (OCT) of the macula reveals a disorganization of retinal architecture related to a sequelae of Retinochoroiditis at the left eye (Figure 3). General examination was unremarkable including the neurological examination. Although we have no serological evidence of congenital infection, we conclude to the diagnosis of macular sequelae secondary to congenital toxoplasmosis based on the following arguments: the presence of a toxoplasmic retinochoroiditis, no acute ocular symptoms, a history of squint in affected eye and a notion of untreated febrile episode during the pregnancy.

## Discussion

Retinochoroiditis is the most common ocular manifestation of congenital toxoplasmosis [1]. The other clinical manifestations of congenital ocular toxoplasmosis were choroidal coloboma, strabismus, nystagmus, ptosis, microphthalmia, cataract and enophthalmia [4]. Epidemiological evidence suggests that most adult disease arises from infection acquired after birth [5]. Much less is known about the prevalence of infection before and after birth in children with toxoplasmic retinochoroiditis. Ophthalmologists are rarely able to distinguish between toxoplasmic retinochoroiditis due to infection acquired before or after birth, unless other clinical or serological indications are present [2].

Knowledge of the relative contribution and severity of infection acquired before and after birth to symptomatic ocular toxoplasmosis in children would inform counselling and the public debate on the relevance of screening programmes for children [6].

Retinochoroidal lesions due to infection before and after birth were indistinguishable. The presence of bilateral, multiple or posterior pole lesions did not distinguish between the two groups, but most children (84%) presenting with acute ocular symptoms had postnatally acquired infection. Children infected before birth were most likely to be detected through abnormal vision screening or ocular appearance. Children infected after birth all presented with acute ocular symptoms. The site of lesion was similar, regardless of when infection occurred [2].

## Conclusion

Retinochoroiditis is the most common ocular manifestation of congenital toxoplasmosis [1]. The education of pregnant women is crucial for the prevention of congenital toxoplasmosis. Untreated, the majority of affected infants will develop chorioretinitis, deafness and/or neurological symptoms [3].
